# Intraoperative evaluation using mobile computed tomography in anterior cervical decompression with floating method for massive ossification of the posterior longitudinal ligament

**DOI:** 10.1186/s13018-017-0515-1

**Published:** 2017-01-19

**Authors:** Toshitaka Yoshii, Takashi Hirai, Tsuyoshi Yamada, Hiroyuki Inose, Tsuyoshi Kato, Kenichiro Sakai, Mitsuhiro Enomoto, Shigenori Kawabata, Yoshiyasu Arai, Atsushi Okawa

**Affiliations:** 1 Department of Orthopaedic Surgery, Graduate School, Tokyo Medical and Dental University, 1-5-45 Yushima, Bunkyo-ku, Tokyo 113-8519 Japan; 2 Section of Regenerative Therapeutics for Spine and Spinal Cord, Graduate School, Tokyo Medical and Dental University, 1-5-45 Yushima, Bunkyo-ku, Tokyo 113-8519 Japan; 3 Saiseikai Kawaguchi General Hospital, Kawaguchi, Japan; 4 Tokyo Medical and Dental University Spine Group, Tokyo, Japan

**Keywords:** Ossification of the posterior longitudinal ligament (OPLL), Anterior decompression, Floating method, Computed tomography (CT), Intraoperative evaluation

## Abstract

**Background:**

An anterior decompression and fusion (ADF) with the floating method is an effective procedure for treating ossification of the posterior longitudinal ligament (OPLL), allowing a direct decompressive effect on the spinal cord. However, the procedure is skill-intensive, particularly in cases of OPLL with a high canal-occupying ratio. In such cases, there are potential risks for insufficient decompression due to the incomplete floating of the OPLL. Here, we introduce an anterior decompression procedure for massive OPLL, using an intraoperative computed tomography (CT) with a mobile scanner gantry for the intraoperative evaluation of the decompression. We further evaluated the outcomes of ADF using mobile CT in comparison with a historical control of ADF without intraoperative CT evaluation.

**Methods:**

Fifty OPLL patients who underwent ADF with the floating method were evaluated in this study: 25 patients with intraoperative CT (CT group) and 25 patients without CT (non-CT group). In the CT group, intraoperative CT scanning was performed before freeing the ossification from the surrounding bone tissues. The reconstructed images were reviewed to evaluate the extent of bone decompression and thinning of the OPLL. After review of the images, further thinning of the OPLL or removal of surrounding bone was performed as deemed necessary, to complete the floating of the OPLL.

**Results:**

Patients’ background was similar between the CT and non-CT group. Operating time tended to be shorter for the CT group. On the postoperative CT, incomplete OPLL floating due to “impingement” between the OPLL and the medial aspect of the pedicle or uncovertebral joint was observed for four patients (16.0%) in the non-CT group, whereas insufficient decompression was not observed in the CT group.

**Conclusions:**

Intraoperative CT imaging was effective to avoid insufficient decompression following ADF with the floating method for massive OPLL. We also consider that the intraoperative three-dimensional imaging is helpful for providing informative feedback to surgeons to improve performance in skill-intensive surgeries such as ADF with the floating method.

## Background

Cervical ossification of the posterior longitudinal ligament (OPLL) is a common degenerative spinal disease that causes neurological dysfunction [1, 2]. In the early stages, the majority of patients with OPLL may not exhibit any neurological symptoms. As OPLL develops in the spinal canal, the spinal cord and nerve roots are compressed from the anterior direction, which results in myelopathy and/or radiculopathy. Minimally symptomatic patients may be treated conservatively; however, patients with progressive neurological disturbances require surgical treatment [3, 4].

The choice of the optimal surgical procedure for the treatment of cervical OPLL is controversial. Posterior decompression, such as laminoplasty, is relatively simple and has a low complication rate [5]. However, the effect of indirect decompression of the spinal cord is limited for patients with massive OPLL [3, 6]. Anterior decompression and fusion (ADF) is theoretically suitable for the treatment of OPLL because it can provide direct decompression to the spinal cord and can stabilize the involved segments [4, 7]. We previously conducted a prospective study comparing ADF and posterior laminoplasty (LAMP) for the treatment of OPLL. We found that ADF is superior for neurological improvement in patients who have massive OPLL with a ≥50% canal-occupying ratio [8]. However, the ventral decompression procedure is complex and technically demanding, particularly in OPLL with a great occupying ratio [9].

Previous studies demonstrated that the use of three-dimensional fluoroscopy is efficacious for intraoperative evaluation of bone decompression in short-segment anterior cervical discectomy and fusion (ACDF) [10]. Recently, high-resolution reconstructed computed tomography (CT) images can be obtained intraoperatively [11] and are useful for the intraoperative evaluation of adequate decompression during technically demanding anterior cervical surgeries. Here, we introduce the procedure of anterior corpectomy and fusion with the floating method for massive OPLL with the support of intraoperative CT images. Furthermore, the surgical results of this technique were compared with a historical control of OPLL cases that underwent ADF without the use of intraoperative CT.

## Materials and methods

This study was approved by an institutional review board. Written informed consents for participation and for publication have been obtained from the participants. Consecutive patients treated with multi-level ADF for cervical myelopathy caused by OPLL at our institution were included. Patients with myelopathy caused by cervical disc herniation, spondylosis, or tumor, patients with a history of previous cervical spine surgery or injury, and patients who had OPLL that extended to the C1/C2 level and thoracic spine with cord compression were excluded. Also, patients with OPLL treated by posterior procedure were excluded. Twenty-five OPLL patients (20 males, 5 females, average age of 63.2 years old; Table 1) who underwent ADF with the floating method using intraoperative CT at our institution between 2012 and 2015 were investigated in this study. The surgical results from the patients who underwent ADF using intraoperative CT (CT group) were compared with 25 consecutive patients (21 males, 4 females, average age of 58.8 years old; Table 1) who underwent ADF without using intraoperative CT before December 2011 (non-CT group).

**Table 1:** Demographics

	Non-CT group (*n* = 25)	CT group (*n* = 25)	*P*
Age	63.5 ± 9.1	58.8 ± 11.4	0.19
Gender (M/F)	20/5	21/4	0.71
Pre JOA score	11.6 ± 1.7	11.4 ± 3.3	0.83
Canal-occupying ratio (%)	48.8 ± 14.4	57.8 ± 17.0	0.06
Levels of fusion	3.3 ± .0.8	3.0 ± 0.6	0.10

Mean ± standard deviation

### Surgical procedures

**Fig. 1:**
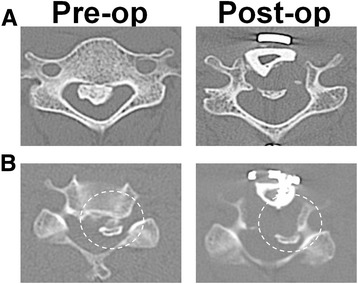
**a** Preoperative (*Pre-op*) and postoperative (*Post-op*) CT (computed tomography) images in the axial plane showed complete floating of ossification of the posterior longitudinal ligament (OPLL). **b** Insufficient floating of OPLL due to the “impingement” between OPLL and medial aspect of the uncovertebral joint

**Fig. 2:**
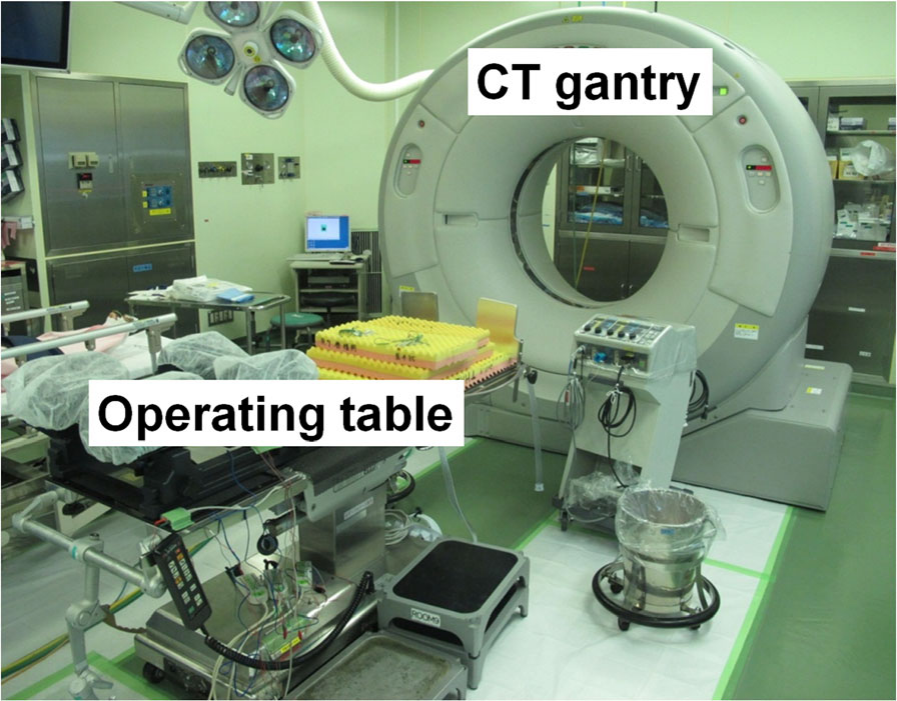
A multidetector CT with mobile gantry in the operating room

**Fig. 3:**
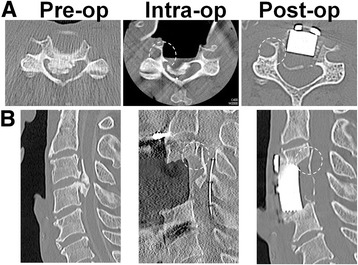
*Pre-op*, intraoperative (*Intra-op*), and *Post-op* CT images in the axial plane (**a**) and sagittal plane (**b**)

We applied the anterior OPLL floating technique described by Yamaura et al. [7, 12] (Fig. 1a). All the surgeries were done by attending spine surgeons certified by the Japanese Society for Spine surgery and related Research. Spinal monitoring during the operation was performed in all patients. A radiolucent operating table made of carbon was used. A standard Smith–Robinson approach to the cervical spine was used [13]. After confirmation and exposure of the appropriate vertebral levels, corpectomy was performed by removing the discs and vertebral bodies. Upon reaching the posterior cortex, drilling was performed carefully under a microscope. The ossification was thinned to the shape of the bottom of a ship until part of the ossification was removed, and the soft tissue underneath the ossification first became visible or felt by the light touch of a microprobe. Before freeing the ossification from the surrounding bone tissues, we took intraoperative CT images to confirm that the width of the decompression and the thinning of the OPLL were appropriate. After careful draping, we moved the helical CT scanner gantry on a floor-embedded rail toward the operating table until the region for imaging was completely within the gantry (Fig. 2). Then, the surgeons moved to a separate room and provided slice plan instructions for scanning to the CT operator, and the cervical spine was scanned using a 16-row multidetector CT unit (TOSHIBA Medical, Tokyo, Japan). The series consisted of 0.5-mm-thick CT sections that were acquired in helical mode and were reconstructed at 0.5-mm intervals. The acquisition parameters were 120 kV and 400 mA. After we had confirmed that the region for imaging was appropriately scanned, the CT gantry was moved away from the operating table, and the CT images were reviewed. In the axial images, we checked the decompression width, position of the vertebral artery and pedicle, approach angle, and thinning of the OPLL (Fig. 3a). In the sagittal images, we evaluated whether decompression was sufficient to reach above or below the spinal cord compression by OPLL (Fig. 3b). This process usually took 10–15 min from the draping to completing the review of the CT images. After review of the images, further thinning of the OPLL or removal of surrounding bone was performed as deemed necessary, to complete the floating of the OPLL. When freeing the ossification from the surrounding bone tissues, the top and bottom ends of the ossification were first cut transversely and then disconnected from the pedicle that was situated laterally. When the thinned OPLL appeared to be like a board floating on water, the decompression of the entire spinal cord was complete. For reconstruction, we used a hydroxyapatite block or a fibular bone graft reinforced by an anterior plate.

### Clinical outcomes

Neurological functions were evaluated using the Japanese Orthopaedics Association (JOA) scoring and associated recovery rate [8]. Additionally, the operation time, intraoperative bleeding, and postoperative complications were evaluated.

## Evaluations of decompression using postoperative CT images

Postoperative CT scans were obtained within 3 months after surgery to assess the decompression using a 64-row multidetector CT unit (TOSHIBA Medical). We evaluated whether the decompression was sufficiently complete in the reconstructed CT images. If the decompression width was insufficient in the axial or sagittal plane, impingement between OPLL and the surrounding bone tissue (the posterior wall of vertebrae, the medial aspect of the pedicle or the uncovertebral joint) could occur and disturb the floating of the OPLL (Fig. 1). The patients were followed for at least 1 year postoperatively.

Statistical analyses were performed using unpaired *t* tests for continuous variables and Fisher’s exact test for categorical data. The significance level was set at *p* < 0.05.

## Results

Neurological disturbance was improved in all the 25 patients; the average improvement rate in JOA score was 65.7%. There was no additional morbidity or progression of neurological symptoms in patients from CT group. Intraoperative reconstructed CT images were successfully obtained from all the patients. Scanning time was usually approximately 60 s. The complete procedure from draping to evaluation of the pictures took less than 15 min. All spinal segments to be decompressed were successfully visualized in axial, sagittal, and coronal views with excellent resolution.

In comparison with the non-CT group, no significant differences were found in comparisons of patient age, gender, preoperative neurological status based on JOA score, and levels of surgery (Table 1). The occupying ratio of OPLL tended to be greater in the CT group (*p* = 0.06), although no significant difference was found. There were no differences in intraoperative blood loss. However, the operating time tended to be shorter in the CT group (Table 2).

**Table 2:** Surgical outcomes in non-CT and CT group

	Non-CT group (*n* = 25)	CT group (*n* = 25)	*P*
Operating time (min)	402.0 ± 125.2	335.9 ± 97.2	0.05
Intra-op blood loss (g)	461.7.2 ± 832.4	358.0 ± 375.6	0.56
Impingement (+)	4 (16.0%)	0 (0%)	0.05
Post-op JOA score	14.9 ± 1.5	15.1 ± 1.5	0.68
IR (%)	64.4 ± 18.8	65.7 ± 19.8	0.82

Mean ± standard deviation

*IR* improvement rate in JOA score

In the non-CT group, incomplete floating of the OPLL was observed for four patients (16.0%) on the postoperative CT images (Table 2). In these patients, impingement between OPLL and the medial aspect of the pedicle or uncovertebral joint that disturbed the complete floating of the OPLL (Fig. 1b) was observed. On the other hand, insufficient decompression was not found in any patients who underwent ADF using intraoperative CT. There were no significant differences in postoperative neurological improvement rate (IR) between the CT and non-CT groups (Table 2).

Eight patients (32.0%) presented with perioperative complications in the non-CT group, including persistent dysphasia in three patients, upper airway obstruction requiring re-intubation in one patient, dislodgement of the graft in two patients, and C5 palsy in one patient. Perioperative complications were observed in only four patients (16.0%) in the CT group, including C5 palsy in two patients, dislodgement of the graft in one patient, and persistent dysphasia in one patient.

## Discussion

The anterior procedure has been widely used for the treatment of cervical degenerative diseases. Generally, for cases in which the compressive pathology is ventral to the spinal cord, an anterior procedure is preferably applied [14, 15]. An anterior approach allows for direct removal of the compressive pathology without the manipulation of the cord. Therefore, ADF is a good option for the treatment of OPLL because it can directly decompress the spinal cord by floating the OPLL and stabilizing the involved segments [7, 16]. Along with others, we have reported that the anterior procedure is particularly efficacious for patients with massive OPLL [8].

The goals of ADF include the removal of neural compression, the restoration of stability, and the restoration or maintenance of spinal alignment [17]. Cooper et al. [18] described that the adequacy of spinal cord decompression is probably the most important factor in determining the outcome. However, anterior decompression for OPLL is sometimes technically demanding, particularly for massive OPLL, which is sometimes accompanied by extensive bleeding during the floating procedure, making visualization of the surgical site difficult. Additionally, massive OPLL often accompanies dural ossification, and thus, there is a high incidence of dural tear and cerebrospinal fluid leakage during decompression that can cause neurological deterioration. In fact, Kimura et al. [9] reported that the size of OPLL is a significant, independent risk factor for perioperative neurological complications in ADF for OPLL patients.

Previous reports indicated that key points for sufficient decompression in the anterior procedure for massive OPLL are adequate orientation, adequate width of decompression, and adequate thinning of the OPLL [7]. If the decompression width is narrow, the edge of the OPLL can impinge the surrounding bone tissue (the posterior wall of the vertebrae, medial aspect of the pedicle or uncovertebral joint), which makes the floating insufficient (Fig 1b). If the thinning of the OPLL is insufficient, floating of the OPLL can be affected by anterior bone graft. Despite these points, the width of the OPLL and the thinning of OPLL are sometimes insufficient because wide decompression and thinning of the OPLL are related to complications such as injury to the vertebral artery and dural tears [19].

In this study, we included patients with massive OPLL (canal-occupying ratio 47.2% in non-CT group and 57.8% in CT group) and found incomplete floating of the OPLL in 16.0% of patients. All of these patients showed an insufficient width of decompression, which disturbed floating of the OPLL. Although none of these patients showed neurological deterioration after surgery and none underwent revision surgery, their improvement rate of neurological score (IR 45.7 ± 12.6%) tended to be lower compared with patients who achieved adequate decompression (IR 66.8 ± 19.4%). Matsuoka et al. [7] also reported that insufficient decompression can cause poor outcomes after surgery and uneven decompression can cause a worsening of neurological symptoms in massive OPLL. We evaluated the CT images before floating the OPLL instead of after floating, because uneven floating of OPLL can cause neurological damage intraoperatively.

Recently, intraoperative imaging technology was developed. Three-dimensional fluoroscopy and navigation systems improve the accuracy of pedicle screw insertion [20]. Cone-beam type CT is also being increasingly used for difficult procedures, such as cervical pedicle screw placement [21]. An intraoperative CT imaging system with a self-moving helical CT scanner gantry can provide intraoperative, high-resolution three-dimensional imaging in the operating room. Since this type of “mobile” CT system was installed in the OR at our institution, we have used this system to assist in cervical surgeries. We previously reported that the evaluation of intraoperative CT images dramatically improved the accuracy of cervical pedicle screw insertion because surgeons could obtain precise orientation [11].

Similarly, intraoperative CT evaluation was effective in anterior decompression with fusion for OPLL. Since the patients in the CT group possessed massive OPLL, decompression was generally difficult because of the above-mentioned reasons. However, insufficient decompression was not found in any patients because we could obtain the precise orientation of the operation during the decompression field by evaluating intraoperative reconstructed images with high resolution. By checking the approach angles to the vertebrae, width and depth of decompression, thinning of the OPLL, and location of the vertebral artery, pedicle, and neural foramen intraoperatively, surgeons can complete decompression easily and appropriately. Navigation system may also be helpful to determine the resected margins intraoperatively. However, in the navigation system, it is difficult to know during surgery how much the surgeon has thinned the OPLL and removed the surrounding bone tissue.

Interestingly, the operating time was shorter for the CT group, although we used approximately 10–15 min for intraoperative CT scanning and the evaluation of images. A potential reason for this decreased operative time is that the surgeon is more certain of their intraoperative orientation and progress, and thus more confident to proceed in these difficult cases. We also considered intraoperative CT evaluation to be effective for lowering the learning curve for surgeons to perform ADF with floating method for OPLL. In this procedure, the surgeon can obtain informative feedback from intraoperative CT images during the completion of the OPLL floating.

Some limitations of this procedure should be noted. Although mobile CT is very useful for obtaining intraoperative high-resolution images, these systems are expensive and may not be available in small hospitals. However, cone-beam CT or three-dimensional fluoroscopy can alternatively be used. We have compared the surgical results in ADF using intraoperative CT with those in the historical control group. Thus, refinement of surgical technique and the benefit to the more recent (intraoperative CT) patients of the surgical learning curve may have an effect on the results. The radiation exposure (approximately 5 mSV: 50 times greater as plane chest X-ray) to patients is another limitation of this technique. Therefore, we perform CT scanning only once during the surgery. To reduce the radiation exposure, we are considering substituting the postoperative CT scan for the intraoperative scan, as no patients in the intraoperative scan group required revision surgery.

## Conclusions

Intraoperative CT scan provided useful information with high-resolution images and was effective in avoiding insufficient decompression and poor neurological outcomes in ADF with the floating method for massive OPLL. We also consider that the intraoperative CT imaging is helpful to provide informative feedback to surgeons performing skill-intensive surgeries such as ADF with the floating method.

## Abbreviations

ACDF: Anterior cervical discectomy and fusion
ADF: Anterior decompression and fusion
CT: Computed tomography
IR: Improvement rate
JOA: Japanese Orthopaedics Association
LAMP: Laminoplasty
OPLL: Ossification of the posterior longitudinal ligament
